# Polymer Nanocomposite Graphene Quantum Dots for High-Efficiency Ultraviolet Photodetector

**DOI:** 10.3390/nano12183175

**Published:** 2022-09-13

**Authors:** Molahalli Vandana, Hundekal Devendrappa, Paola De Padova, Gurumurthy Hegde

**Affiliations:** 1 Department of Chemistry, CHRIST (Deemed to Be University), Hosur Road, Bangalore 560029, India; 2 Centre for Advanced Research and Development (CARD), CHRIST (Deemed to Be University), Hosur Road, Bangalore 560029, India; 3 Department of Physics, Mangalore University, Mangalagangothri 574199, India; 4 Consiglio Nazionale delle Ricerche, CNR-ISM, Via Fosso del Cavaliere, 00133 Rome, Italy; 5 INFN-Laboratori Nazionali di Frascati, Via Enrico Fermi Frascati 40, 00044 Rome, Italy

**Keywords:** polypyrrole, graphene quantum dots, excitation spectra, novel UV photodetector, ionization potential, electron affinity

## Abstract

Influence on photocurrent sensitivity of hydrothermally synthesized electrochemically active graphene quantum dots on conjugated polymer utilized for a novel single-layer device has been performed. Fabrications of high-performance ultraviolet photodetector by depositing the polypyrrole-graphene quantum dots (PPy-GQDs) active layer of the ITO electrode were exposed to an Ultraviolet (UV) source with 265 and 355 nm wavelengths for about 200 s, and we examined the time-dependent photoresponse. The excellent performance of GQDs was exploited as a light absorber, acting as an electron donor to improve the carrier concentration. PGC4 exhibits high photoresponsivity up to the 2.33 µA/W at 6 V bias and the photocurrent changes from 2.9 to 18 µA. The electrochemical measurement was studied using an electrochemical workstation. The cyclic voltammetry (CV) results show that the hysteresis loop is optically tunable with a UV light source with 265 and 355 nm at 0.1 to 0.5 V/s. The photocurrent response in PPy-GQDs devices may be applicable to optoelectronics devices.

## 1. Introduction

In the past few years, Graphene quantum dots (GQDs) have attracted significant attention due to their unique characteristics such as highly tunable photoluminescence (PL) [1], good solubility in water, superior biocompatibility [2], large-scale production, and low cost. GQDs are one of the most significant zero-dimensional materials due to their prominent electronic [3] and optical properties [4]. A unique characteristic of the GQDs is the formation of many layers and their size being less than 30 nm. GQDs also drew attention to optoelectronic [5] applications due to their exemplary fluorescence behavior, charge induced by the quantum confinement effect [6], and edge effect [7,8]. The evolution of quantum dots dispersion in the polymer matrix is an important aspect that is significantly studied. Embedding quantum dots in polymeric matrices can enhance their stability and end agglomeration too. It shows wide absorption spectra ranging from UV to visible wavelengths. Furthermore, GQDs are expected to show better biocompatibility than other inorganic semiconductor nanoparticles. Hence, it is a motive to study the photoresponsive properties in the conducting polymer-graphene quantum dots nanocomposites [9]. Among various conducting polymers, polypyrrole (PPy) is a unique property with simple synthesis, low cost, mass production, high electrical conductivity, and high charge transfer resistance. It is a conjugated polymer with strong absorption in the visible region and is environmentally stable. However, polypyrrole applications are limited due to poor processability, mechanical properties, and solubility [10,11]. The modification of conductive polymer with carbon-based material is essential because the carbon material shows improved mechanical, electrical, and electrochemical properties compared with the conducting polymers alone, leading to a wide variety of applications, including sensors, catalysis, and energy storage. Inorganic colloidal quantum dots have higher stability under ambient conditions, good optical properties, and great potential of composites that have demonstrated high-performance UV photodetectors at a low cost with facile fabrication. Based on the work by W. Xu et al. [12] proposed that the photodiode based on ZnO-GQDs/PVA nanocomposite shows a 0.06 s short rise time with a responsivity of 46.5 A/W. Eunhee Park et al. [13] presented the Poly (9-vinyl carbazole)/SnO_2_ quantum dot heterojunction-based self-powered high-performance ultraviolet photodetector. The device demonstrated exceptional responsiveness (49.6 mA W1 at 254 nm and 166 mA W1 at 220 nm) and detectivity (2.16 1010 Jones at 254 nm) under optimal conditions. A hybrid photodetector constructed by the poly (3-hexylthiophene-2,5-diyl) (P3HT) bulk heterojunction (BHJ) composite is blended with the photoactive layer of lead sulfide QDs (PbS QDs) to produce large interfacial charge separation resulting in improved photocurrent of the hybrid photodetector. A high-performance hybrid photodetector of CuInSe2 nanocrystal and poly (3-hexylthiophene) exhibits a detectivity greater than 1010 cm Hz/W [14]. Dongyang Zhu et al. [15] proposed recent developments in infrared photodetectors based on polymers. Polymer materials are viable for a new generation of IR PDs due to their designable molecular structure, solution processability, large-area preparation, and inherent flexibility. The performance of PDs is improved, and their application scenarios are broadened by various molecular design methodologies and cutting-edge device designs, which further encourages the rapid development of IR PDs. Baosen Zhang et al. [16] reported that broadband PDs based on perovskites that have been solution-processed at ambient temperature and feature highly electrically conductive PbSe quantum dots (QDs). The solution-processed BHJ broadband PDs exhibit responsiveness of 10 mA/W, a detectivity of 1011 Jones, and a linear dynamic range of 53 dB in the spectral response ranging from 350 nm to 2500 nm.

In this work, we demonstrate a facile, low-cost synthesis in which the PPy-GQD composite can be prepared through a chemical oxidation polymerization process. It is essential to make a strategy to synthesize suitable size of graphene quantum dots, and uniform distribution to address the current challenges because these features play a significant task in good reversibility and response speed. The luminescence of the GQDs was found to be tunable by varying the concentration. Furthermore, the electrical hysteresis behavior of the composite was observed. The hysteresis effect of the device can be tuned by the concentration and size of the GQDs. We report a novel UV photodetector fabricated from PPy-GQDs composites. Because of the large band gap of GQDs, the devices could detect UV light with wavelengths of 265 and 355 nm at 0.1 to 0.5 V/s. This work may open a wide range of applications for GQD composite-based light-sensitive devices.

## 2. Materials and Methods

### 2.1. Synthesis of Graphene Quantum Dots

The graphene quantum dots were prepared by the one-step hydrothermal method. Where 3 wt% of glucose powder was dissolved in 100 mL of deionized water, and it was sonicated for 20 min after complete dissolution [17]. This mixture was poured into 100 mL of Teflon-lined stainless steel autoclave and heated at 180 °C for 3 h; the resultant product has changed its color from transparent to pale yellow. The as-prepared solution was centrifuged at 3000 rpm for 30 min, and the resulting yellow solution is graphene quantum dots.

### 2.2. Synthesis of Polypyrrole-Graphene Quantum Dots Composite

A 0.3 M of pyrrole monomer and 0.7 M FeCl_3_ solution was mixed in 100 mL of 1 M HCl with continuous stirring. The 20 and 40 mL of GQDs were added to the above solution; the solution was heated at 60 °C for 20 min. 100 mL of 0.7 M FeCl_3_ was poured into the above solution and kept for polymerization (0 °C for 24 h), centrifuged at 15 min, and PPy-GQDs (PGC) composites were obtained. The 20 and 40 mL graphene quantum dots present in the polypyrrole were coded as PGC2 and PGC4.

### 2.3. Fabrication of a Single Layer Device

**Scheme 1:**
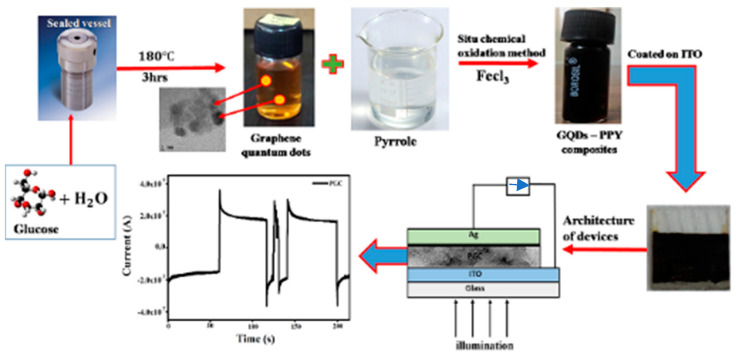
Schematic diagram of the synthesis of PPy-GQDs composites and fabrication of a single-layer device.

The ITO-coated glass substrates were cleaned ultrasonically in acetone and methanol at 27 °C for 10 min and were rinsed in deionized water. The chemically-cleaned ITO-coated glass substrates were dried by using N_2_ gas with a purity of 99.9999%. The PPy-GQDs is an active layer deposited by simple brush coating on ITO, dried at 60 °C for 15 min. The silver paste was applied by a doctor blade method on the top of the device with an effective area of 1 cm^2^. The fabricated device having a thickness of 91.3 nm (PGC2) and 84.7 nm (PGC4) was examined for CV and IT measurement under UV light. The scheme of the PPy-GQDs synthesis and fabrication of the device is cited in Scheme 1.

## 3. Results

We report in the following sections several measurements, such as FT-IR, XRD, UV-Visible absorption, PL, cyclic voltammetry, TEM, device photoresponse and photocurrent, Mott Schottky plot, and finally energy level diagram, which serves to highlight the physical and chemical properties of the synthesized composite nanostructure GQDs-based and UV photodetector device.

### 3.1. FT-IR Study

First, we report the FT-IR spectra of the PPy showing the characteristic peak position at 1044 cm^−1^ attributed to the C-N bond. The peaks observed at 1530 cm^−1^ and 3732 cm^−1^ are associated with the pyrrole ring’s C-C and N-H stretching vibration [18]. In the composite, these two peaks have been shifted to 1539 cm^−1^ and 3749 cm^−1^ due to the interaction between the carboxylic acid group of GQDs and the N-H peptide bond of PPy.

**Figure 1:**
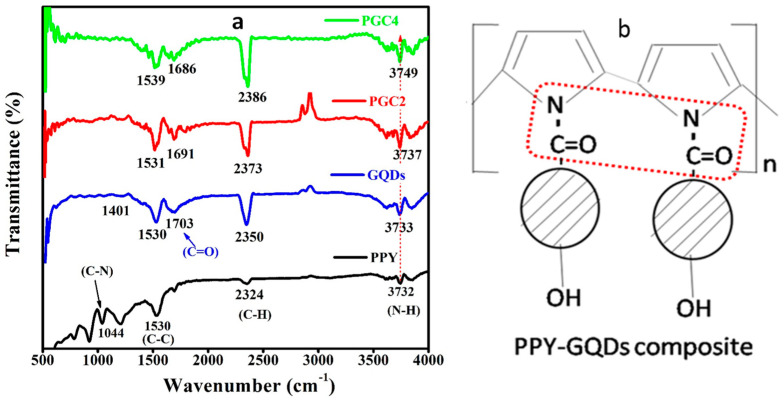
FT-IR spectra of Polypyrrole, Graphene quantum dots, PGC2, PGC4 composites (**a**), and proposed chemical interaction in PPy-GQDs composite (**b**).

A peak at 2324 cm^−1^ corresponding to the C-H bending vibration shifted to 2386 cm^−1^ after the addition of GQDs. The C=O bond observed at 1703 cm^−1^ is attributed to the characteristic GQDs peak and wave number reduced to 1691 and 1686 cm^−1^ as well as decreasing the intensity of peaks in PGC2, and PGC4 composites noticed the GQDs dispersion in PPy system, it is not seen in PPy as shown in Figure 1 and has been reported as a characteristic peak of GQDs [19]. Interestingly, this peak downshifted to 1686 cm^−1^ due to the π-π interaction between the GQDs layer and aromatic PPy ring, elucidated that the interaction of C=O with N-H of polymer, the most probable chemical reaction mechanism, is that shown in Figure 1b. In addition, the peak at 1401 cm^−1^ is attributed to the skeletal vibration from the graphene domain [20]. Therefore, all these peaks confirmed the PPy-GQDs composite.

### 3.2. XRD Study

**Figure 2:**
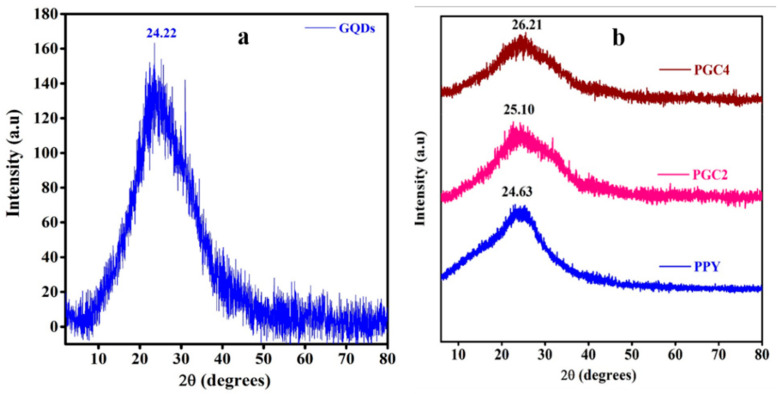
XRD study of (**a**) GQDs, (**b**) PPy, PGC2, and PGC4.

The X-ray diffraction analysis of PPy and PPy-GQDs composites shown in Figure 2 elucidate this point. The GQDs exhibit (002) peak related to the interplanar spacing of 0.23 nm, which is less than the 0.33 nm, the interplanar spacing of graphite (Figure 2a). When GQDs are incorporated into the polypyrrole matrix, the diffraction peak of GQDs at 24.22° (Figure 2a) overlapped with the 24.63° (Figure 2b) amorphous peak of PPy, which resulted in the broad and intense peak at 25.10° shifted to 26.21° with a greater concentration of GQDs.

The peak shifted to a higher diffraction angle and increased the broadness with an intensity, which means that the crystalline nature of GQDs affects the amorphous structure of PPy and indicates homogenous dispersion of GQDs onto the PPy matrix [21]. The average chain separation [22] of PPy, GQDs, PGC2, and PGC4 is 4.51 A^0^, 4.58 A^0^, 2.26 A^0^, and 2.17 A^0^, respectively.

### 3.3. UV-Visible Absorption Study

**Figure 3:**
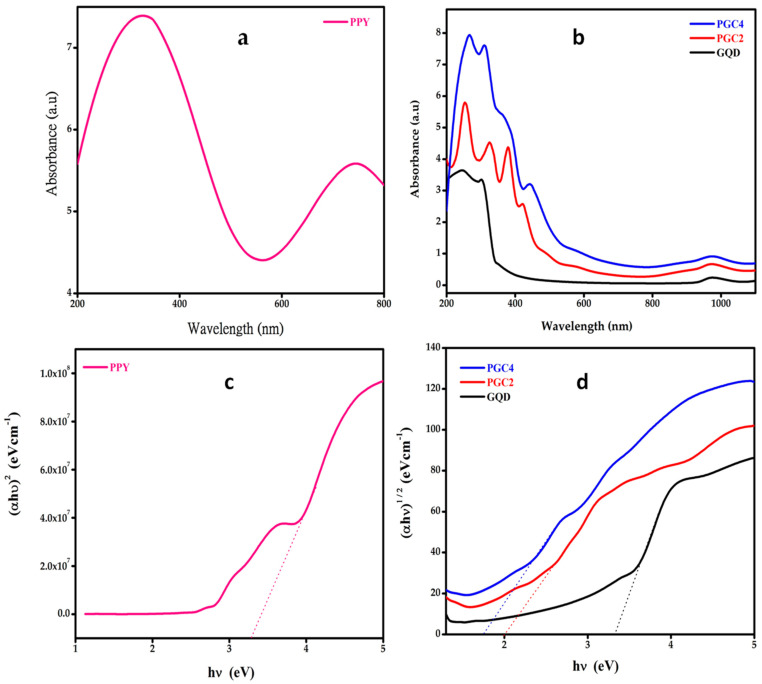
UV-Visible spectra of (**a**) PPy. (**b**) GQDs, PGC2, and PGC4 composites and (**c**) Tauc Plots of (αhν)^2^ versus hν for the determination of direct bandgap (E_gd_) of PPy. (**d**) Tauc Plots of (αhν)^2^ versus hν for the determination of direct bandgap (E_gd_) of GQDs, PGC2, and PGC4.

To characterize the optical features of PPy, GQDs, and PGC composites, their UV-Visible absorption spectra are shown in Figure 3. The UV-Visible spectra of the PPy peak observed at 330 nm are ascribed to the π-π* bipolaron transition (Figure 3a). A typical absorption peak of GQDs at 242 nm is due to the π-π* transition of the aromatic sp^2^ domain, and other peaks at 302 nm are attributed to the n-π* transition of C=O. The characteristic peak 242 nm of GQDs has enhanced the intensity and appeared two new peaks 329 and 379 nm in the PGC2 composite due to GQDs confinement effect and oxygen of GQDs interact with the hydrogen of PPy; as a result, promotion of n-π* transition [23]. The PGC4 composite shows a peak at 255, 329 nm (Figure 3b) derived from the π-π* and n-π* transition of GQDs, and the shift of the peak at 423 nm is attributed to the conjugation between the GQDs and PPy [24]. Figure 3c shows the optical absorption is calculated using the Tauc equation αhν = A (hν − E_g_) n [25], where E_g_ is bandgap, α is the absorption coefficient, ν is frequency, A is constant, and depending on the mode of transition, n takes the different values. Here n = 0.5 shows the best fit for optical absorption data. Therefore, amazingly, the material allows the important direct bandgap transition. A plot of (αhν)^2^ versus hν (Figure 3d) and extrapolating the linear portion of the graph on the hν axis gives that the bandgap values are 3.4 eV, 3.28 eV, 2.05 eV, and 1.62 eV for PPy, GQDs, PGC2, and PGC4, respectively.

Thus, the electrical conductivity of polypyrrole increases in the presence of GQDs, due to a decrease in the bandgap [26]. To visualize the morphology of the realized composite nanostructures, we show in the next paragraph the impressive FE-SEM images, which clarify the GQDs allocation sites.

### 3.4. FE-SEM Analysis of PPy and the PPy-GQDs Composite

**Figure 4:**
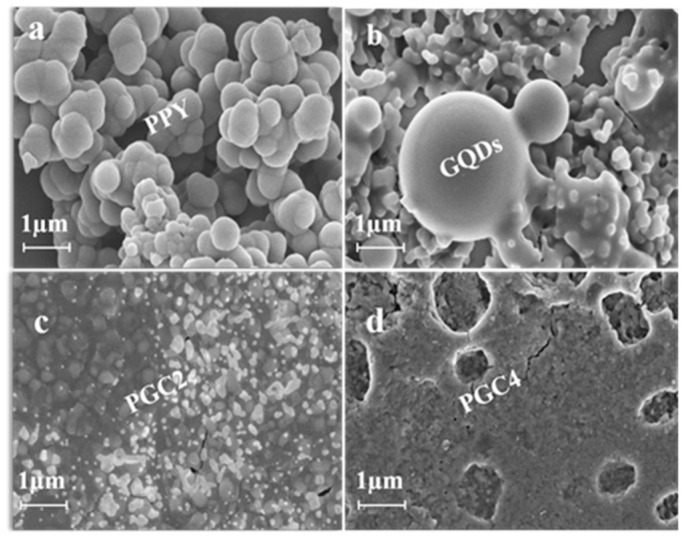
FESEM images of (**a**) PPy, (**b**) GQDs, (**c**) PGC2, and (**d**) PGC4.

The pure PPy FE-SEM images show massive spherical morphology due to their physical fusing or polymerization among the chains, as shown in Figure 4a. The FESEM of GQDs shows the spherical shape morphology shown in Figure 4b. Figure 4c,d, shows the composite morphology of PGC2 and PGC4, respectively, demonstrate the porous structure [27].

The GQDs initially had infinite oxygen groups such as –COOH and –OH. By increasing the concentration of GQDs, it was unable to provide enough templates for the growth of the pyrrole monomer on its surface [28]. Thus, it is clear that GQDs are embedded in the porous region and flatten the surface of the polymer matrix.

### 3.5. PL Analysis of PPy, GQDs, and PPy-GQDs Composite

**Figure 5:**
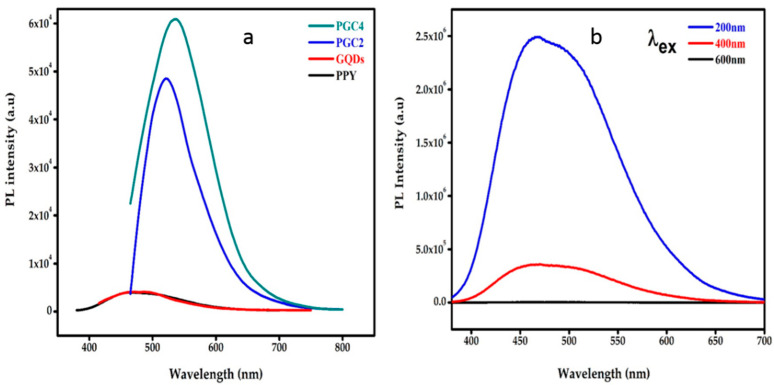
Photoluminescence of the (**a**) PPy, GQDs, and PGC composites (PGC2, PGC4) for the same excitation wavelength. (**b**) PL spectra of pure GQDs at various excitation wavelengths (λ_ex_).

The photoluminescence spectra of PPy, GQDs, and PPy-GQDs composite are shown in Figure 5. The excitation wavelength (λ_ex_) is 400 nm for GQDs, and the PL peak position of GQDs is found at 462 nm. The PL peak of PPy is at 467 nm, and PGC composites are 523 and 535 nm for PGC2 and PGC4 composites with the same excitation wavelength, respectively, as shown in Figure 5a.

The redshift behavior of the composite was attributed to the strong orbital interaction between the π-conjugated PPy and GQDs [29]. The electron transfer that takes place from polymer to GQDs may appear narrow bandgap, causing the tuning of PL by varying the concentration of GQDs shown in Figure 5b. As increasing the concentration of GQDs, the PL peak moves towards the longer wavelength side from 523 to 535 nm, as shown in Figure 5a, mainly due to the functionalization of GQDs in the PPy matrix. Pure GQDs exhibit exciton-dependent emission with a change in the excitation wavelength from 200 to 600 nm gradually increasing the emission peak intensity [17,30], as shown in Figure 5b. The exciton-dependent PL shift attributed to the inhomogeneities of the energy level originates from the size of GQDs.

### 3.6. The Cyclic Voltammetry Study

**Figure 6:**
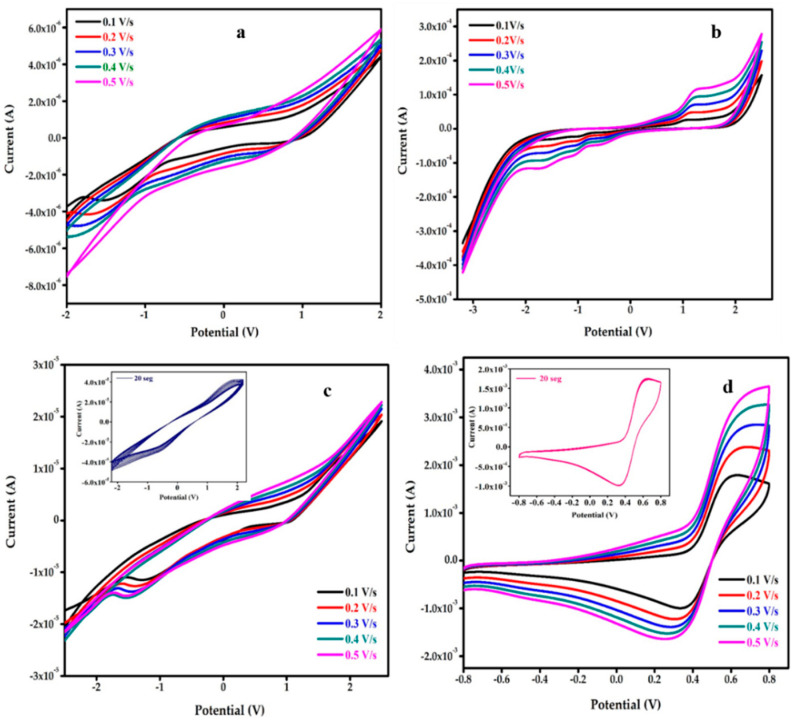
CV curves of (**a**) PPy, (**b**) GQDs. (**c**) PGC2 composite (**d**) PGC4 composite at different scan rates 0.1 to 0.5 V/s. The inset is the CV curves of composites at 20 segments at a 0.1 scan rate.

The electrochemical behavior of PPy, GQDs, and the PGC composite was studied by CV measurement shown in Figure 6a–d. In a three-electrode system, platinum was used as a counter electrode, Ag/AgCl as a reference electrode, and PGC composite as a working electrode with 1 M NaCl solution as the electrolyte. A working electrode was prepared by coating the prepared materials on ITO glass (with a 1 cm^2^ area) and drying at 60 °C for 1 h.

The potential range of −2 to 2 V for PPy, −3 to 3 V for GQDs and PGC2, and −0.8 to 0.8 V for PGC4 composite at different scan rates were used here. As the scan rate increases from 0.1 to 0.5 V/s, the corresponding current increases, suggesting that the current is directly proportional to the square root of the scan rate. The CV curves of the PGC4 composite show a rectangular shape confirms the good electrochemical behavior.

### 3.7. Transmission Electron Microscopy

**Figure 7:**
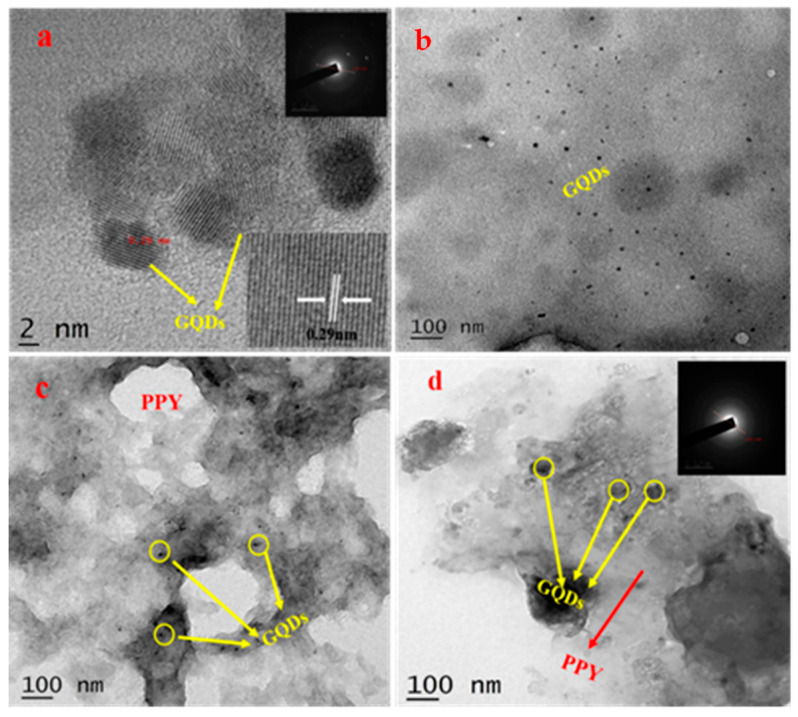
(**a**) HR-TEM image of GQDs. (Inset: HRTEM image of single graphene quantum dots and corresponding SAED pattern) (**b**) The TEM image of GQDs. (**c**) TEM image of PGC2, (**d**) PGC4 composites (inset: SAED pattern of PGC4).

The HR-TEM image of individual GQDs has a lattice fringe of 0.29 nm (Figure 7a), which corresponds to the [110] basal plane distance of the bulk graphite [31,32]. The average particle size observed in GQDs is 2 to 7 nm. Due to the high pressure in the hydrothermal technique, the carbonization of glucose first occurs, then it nucleates, crystallizes, and grows [33]. In the PGC composite, the pyrrole radical cation is polymerized on the surface of GQDs to form the island-like matrices in the composites, as shown in Figure 7c,d. The HR-TEM image shows an almost homogeneous distribution of GQDs in the polymer matrix, but compared to pure GQDs, the shape of GQDs in the polymer is irregular due to the chemical reaction between the functional group located at the GQDs surface and the polymer matrix [34]. The SAED images of GQDs and PGC4 are shown in inset Figure 7a,d, representing a well-defined diffraction pattern that confirmed the crystalline structure of GQDs. However, there was no such diffraction pattern for the composite due to the amorphous nature of PPy, which correlates with the XRD result.

### 3.8. Photoresponse of PPy-GQDs Composite Device

The CV measurement on UV photodetectors under the illumination of 265 and 355 nm UV light at a power of 8 W and the dark region characterized the device’ss performance. The holes located at GQDs are shifted to the ITO electrode, enhancing photodetector performance. The attachment with polymer with the GQDs layer might facilitate the enhancement in the photoresponse of PDs [17]. In most cases, the photocurrent decreased slightly compared to the dark current due to the absorption of oxygen and water molecules on the surface of the graphene quantum dots in the air. When the device is enclosed in the dark, absorbed oxygen and water molecule are ionized by capturing the free electron from GQDs due to their strong electronegativity. The current was measured as a function of voltage swept from −4 to +4 V. Under the same voltage and scan rate, PPy-GQDs produce a higher hysteresis sign of more energy storage capacity [35]. The area calculates the stored energy under the hysteresis loop, which is calculated to be 8.2 nA and 2.3 × 10^−5^ A for PGC2 and PGC4, respectively, in the scan rage ±4 V. It is evident that more than one order of magnitude energy stored in PGC4 compared to PGC2.

**Figure 8:**
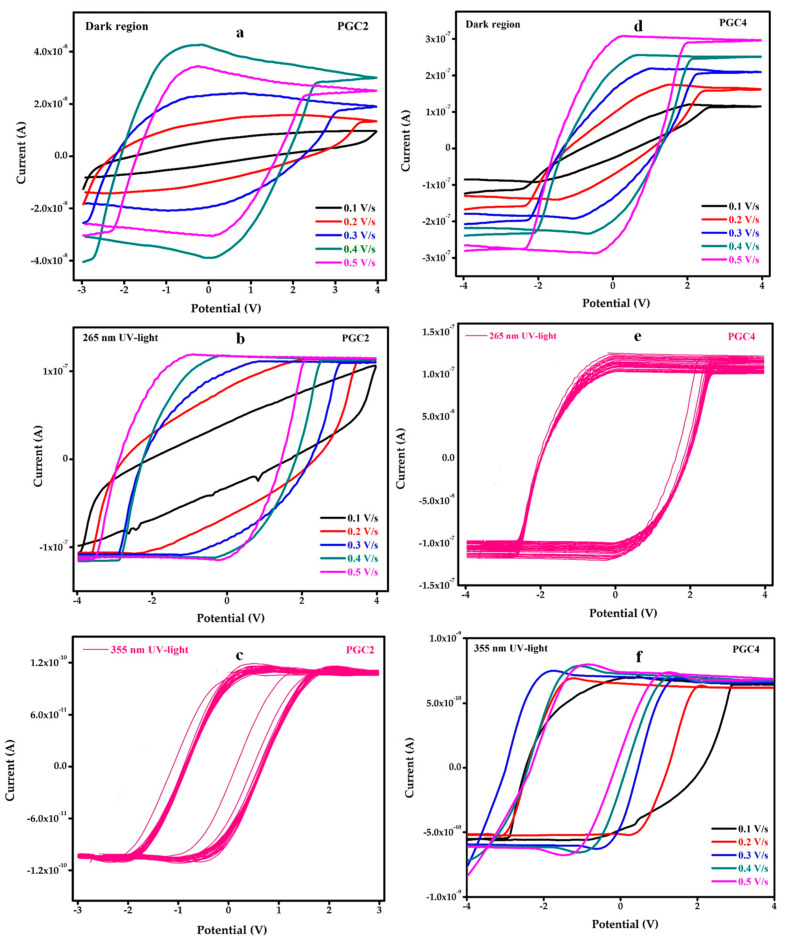
Typical CV curves of the PGC2 composite devices measured in the (**a**) dark region, (**b**) under 265 nm UV light, and (**c**) under 355 nm UV light. CV curves of PGC4 composite devices measured in the (**d**) dark region, (**e**) 265 nm UV light, and (**f**) under 355 nm UV light.

Typical CV curves of the PGC2 composite devices were measured in the Figure 8a dark region, Figure 8b under 265 nm UV light, and Figure 8c under 355 nm UV light. CV curves of PGC4 composite devices were measured in the Figure 8d dark region, Figure 8e 265 nm UV light, and Figure 8f under 355 nm UV light. As the scan rate increases from 0.1 to 0.5 V/s, the corresponding current rate was found to increase, which means that the current is directly proportional to the square root of the scan rate [14].

Typically, the influence of the temperature on photodetection varies as the square of temperature, as reported in Ref. [36]. Thus, the change in temperature affects the photodetector more in photovoltaic mode, than in the photoconductive mode of operation. In general, in the photo-conductive mode of operation, the dark current may approximately double for every 10 °C increase in temperature. As the temperature continuously rises, after 250 °C the photocurrent of a device suffers degradation.

#### Photocurrent Measurements

GQDs under illumination with UV light generated electron-hole pairs. The functionality of the photodetector was investigated by plotting the I-V curve to determine the photocurrent,

I_ph_ = _Ilight_ − _Idark_ (1)

Here I_dark_ & I_light_ are the dark current measured and the current under light illumination.

The spectral response of the photodetectors was investigated under the illumination of an 8 W light UV source with wavelengths of 265 and 355 nm by Equation (2)

**Figure 9:**
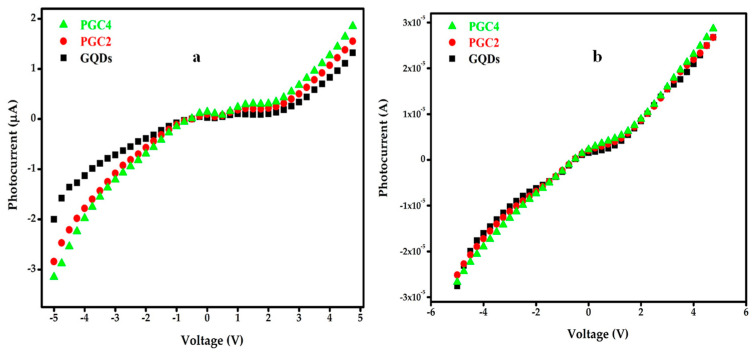
A comparison between the photocurrents of three photodetector devices under the illumination of (**a**) 265 nm & (**b**) 355 nm UV light.

R = I_ligh_t − I_dark_/(P_inc_) (2)

where P_inc_ is the incident illumination power in the effective area [37]. We have compared the photoresponse of GQDs, PGC2, and PGC4 under 265 nm and 355 nm illumination, as shown in Figure 9a,b. The amount of responsivity of about 0.33 µA/W & 0.36 µA/W at the wavelength of 265 nm UV-light is measured for PGC2 and PGC4 composite. The corresponding photocurrent is 2.65 µA and 2.9 µA. At 360 nm wavelength of UV-light, both PGC2 and PGC4 exhibit photocurrents of 15.5 µA & 18.7 µA, corresponding responsivity is 1.93 & 2.33 µA/W. At both wavelengths of UV light, the PGC4 produces a higher photocurrent than PGC2 under the same light intensity and bias voltage. The amount of responsivity is calculated from Equation (2) [38]. The responsivity of the PGC4 device is about 2.33 µA/W, and the PGC2 device is about 0.36 µA/W. Based on the obtained result highest 18.7 µA photocurrent recorded for the PGC4 photodiode, it is observed that the quantum dots in the polymer enhanced the electron mobility as a result of the high electrical characteristics of the nanocomposite.

**Figure 10:**
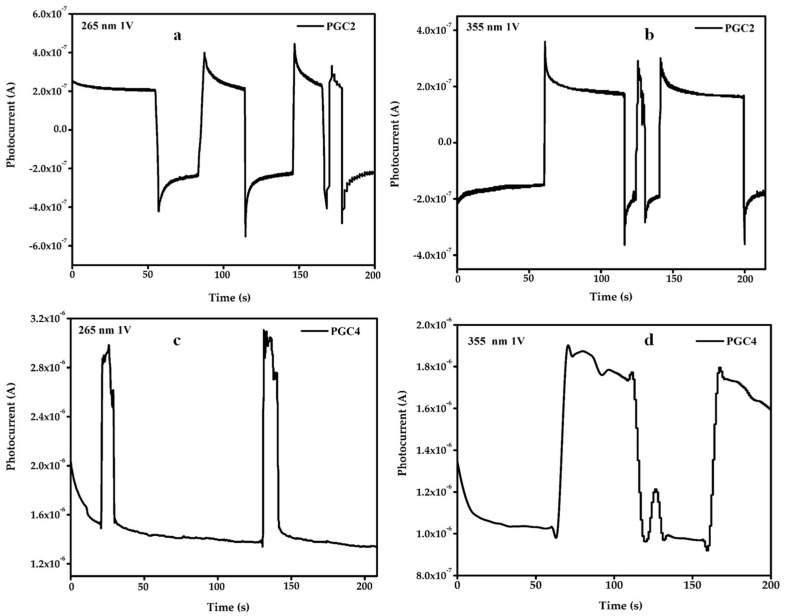
The time response of the PGC2 devices with the symmetric electrode, (**a**) 265 nm UV light (**b**) 355 nm UV light, and PGC4 device with (**c**) 265 nm UV light and (**d**) 355 nm UV light.

An essential parameter for the photodetection of PGC2 and PGC4 is the response time, analyzed in Figure 10. Both composites’ photoresponse enhanced the response time and responsivity of the PPy-GQDs composite, ascribed to the improved interconnection between GQDs and PPy. The UV light sources with 265 nm and 355 nm were illuminated. The photocurrent of both the composite measured at a different wavelength, but for superior comparison responsivity of PGC4 is 2.33 µA/W at 355 nm illumination is a higher order of magnitude than 265 nm illumination is mentioned in Table 1.

### 3.9. Mott Schottky Plot for PGC2 and PGC4 Composites

**Figure 11:**
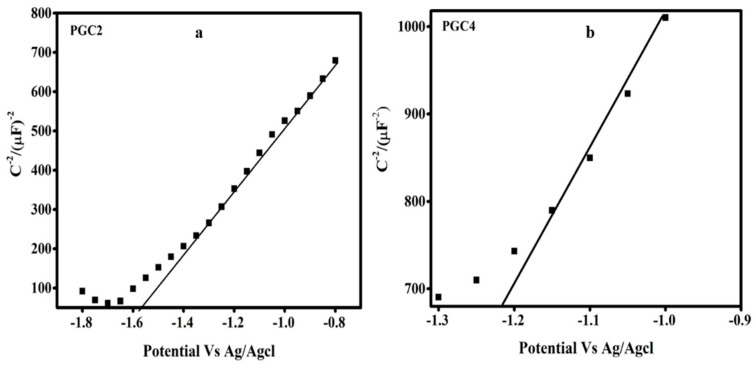
M-S Plot for PGC2 (**a**) and PGC4 (**b**) composite.

The Mott-Schottky plots are performed to study the electronic band gap and the flat band potential of the PGC2 and PGC4 composite. The plot of Figure 11a shows the semiconductor’s flat-band potential (EFB), which is necessarily applied to the SEM.

The PGC2 and PGC4, EFB values are −1.58 (Figure 11a) and −1.23 V, (Figure 11b) and the charge carrier’s transfer interface is shown in the M-S Plot.

### 3.10. Energy Level Diagram of PGC Composite Devices

**Figure 12:**
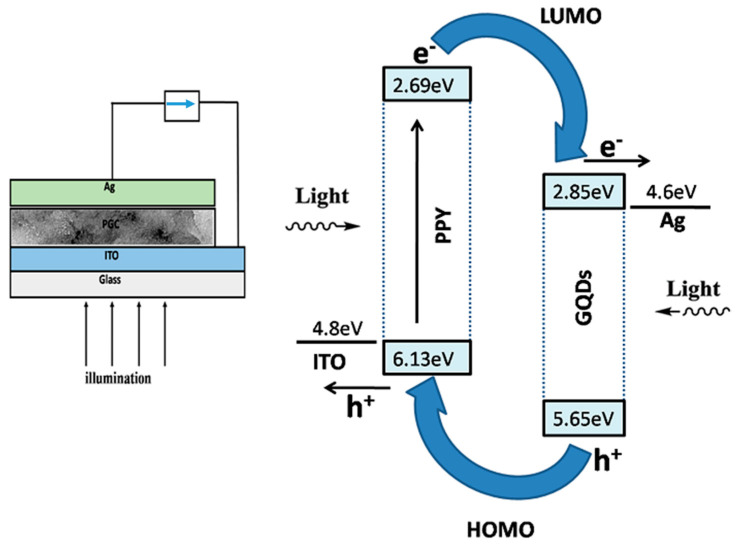
A scheme of photodetector device and the energy level diagram of PPY and GQDs by the illumination of light.

The working mechanism of the PGC composite can be understood with the help of an energy band diagram. Figure 12 shows a schematic diagram of the energy band mechanism of the electrons and holes during UV illumination for the device. When illumination of UV light is used, the PDs through the ITO electrode, the photons penetrate the PPy-GQDs layer (active layer) exhibit the exciton creation. When photogenerated exciton diffused into both polymer and quantum dots composite while electron move to Ag layer resulting generation of photocurrent [39,40].

E_LUMO_ = [(E_red_ − E_1/2_ (ferrocene)) + 4.8] eV (3)

E_HOMO_ = [(E_oxi_ − E_1/2_ (ferrocene)) + 4.8] eV (4)

E_g_ = E_LUMO_ − E_HOMO_ (5)

The energy level in E_HOMO_ is 6.13 eV, E_LUMO_ is 2.69 eV, and the electrochemical bandgap is 3.44 eV. Similarly, the electrochemical bandgap (E_g(el)_) obtained for PGC2 and PGC4 is shown in Table 2.

Bredas et al. [41] reported the relation, onset of oxidation and reductions with the ionization potential and electron affinity values,

[E onset] ox = IP − 4.4(6)

[E onset] red = EA − 4.4(7)

E_g_ = IP − EA (8)

The polymer attains a high electrical conductivity, low ionization potential, and a larger electron affinity. Graphene quantum dots have a strong π-conjugated bond showing large orbital interaction between pyrrole monomer, and the π-conjugated system elevates primary HOMO to a higher energy orbit [42]. The bandgap of PGC4 is smaller than that of PGC2, and the device exhibits good optical properties and great potential of composite, demonstrating high performance and a low-cost UV PDs device. We can remark that any kind of degradation, with respect to photoresponsivity measurements, was observed in the PPy-GQDs composite samples stored in a vacuum-sealed desiccator at room temperature for 3 years.

Table 3 demonstrates the improved photoresponse of PGC2 and PGC4 compared to other results due to the enhanced interconnection of GQD by the island-like polymer matrices, which facilitates carrier transport within the polymer matrices. The photocurrent switching phenomenon in GQD and PPy-GQDs devices may open up novel applications in optoelectronics.

## 4. Conclusions

In summary, highly luminescent GQDs were synthesized by the hydrothermal method. A chemical oxidation polymerization technique is used to synthesize PPy-GQDs composites. The FT-IR results specified the chemical interaction and the crystal fringes in the TEM image confirmed the homogeneous dispersion of GQDs in the polypyrrole surface. The PL confirmed that the redshift of the composite is ascribed to π-conjugation interaction b/w PPy and GQDs. The novel photodetector exhibits very high responsivity 2.33 µA/W in PGC4 composite for 355 nm UV light. The improved responsivity compared to PGC2 (1.93 µA/W) resulted in the reduction of the carrier transportation barrier, giving rise to excellent stability and reproducibility, fast response speed, and highly durable device expanding great opportunities by using PPy-GQDs composites in high-performance, low-cost UV photodetectors. Further work will continue to establish the stable monolayer of active material in the hybrid photodetector device for different wavelengths.

## Figures and Tables

**Table 1:** Summary of the photoresponse at 1 V for PPy-GQDs composite devices with varying thickness.

Device	Thickness (nm)	I_ph_ at 265 nm (µA)	R at 265 nm (µA/W)	I_ph_ at 355 nm (µA)	R at 355 nm (µA/W)
PGC2	91.3	2.65	0.33	15.5	1.93
PGC4	84.7	2.9	0.36	18.7	2.33

**Table 2:** Electrochemical study of the PPy, GQDs, and PGC composites.

Sample	HOMO (eV)	LUMO (eV)	E_g(el)_ (eV)	E_g(opt)_ (eV)	I_p_ (eV)	E_A_ (eV)
PPy	6.13	2.69	3.44	3.4	6.14	2.7
GQDs	5.65	2.85	2.8	3.28	5.66	2.86
PGC2	5.29	3.08	2.21	2.05	5.3	3.09
PGC4	5.14	3.66	1.48	1.62	5.15	3.67

**Table 3:** A summary of the photoresponse comparison of previously published papers with our results.

Device	Illumination	Photocurrent (I_ph_)	Ref
Hybrid polymer-ZnO quantum dot	365 nm light at 0.4 V	10^8^ A	[35]
Single-Walled Carbon Nanotube–CdS Nanocomposites	380 nm light	6.2 × 10^−6^ A	[43]
PANI-GQDs	532 nm light at 3 V	23 × 10^−9^ A	[44]
P3HT: CISe hybrid film	7.63 mW cm^−2^ light at 0.4 V	15 × 10^−12^ A	[14]
P3HT: PC_61_BM composites	500 nm at 1 V	10^−9^ A/cm^2^	[12]
PGC2	355 nm light 1 V	15.5 × 10^−6^	This work
PGC4	355 nm light 1 V	8.7 × 10^−6^	This work

## Data Availability

MDPI Research Data Policies.
